# Clustered surface amino acid residues modulate the acid stability of GH10 xylanase in fungi

**DOI:** 10.1007/s00253-024-13045-1

**Published:** 2024-02-16

**Authors:** Yanwei Xia, Wei Wang, Yaning Wei, Chuanxu Guo, Sisi Song, Siqi Cai, Youzhi Miao

**Affiliations:** Jiangsu Provincial Key Lab for Organic Solid Waste Utilization, National Engineering Research Center for Organic-Based Fertilizers, Jiangsu Collaborative Innovation Center for Solid Organic Waste Resource Utilization, Nanjing Agricultural University, Nanjing, 210095 China

**Keywords:** Acid stability, GH10 xylanase, Thermostability, Filamentous fungi

## Abstract

**Abstract:**

Acidic xylanases are widely used in industries such as biofuels, animal feeding, and fruit juice clarification due to their tolerance to acidic environments. However, the factors controlling their acid stability, especially in GH10 xylanases, are only partially understood. In this study, we identified a series of thermostable GH10 xylanases with optimal temperatures ranging from 70 to 90 °C, and among these, five enzymes (Xyn10C, Xyn10RE, Xyn10TC, Xyn10BS, and Xyn10PC) exhibited remarkable stability at pH 2.0. Our statistical analysis highlighted several factors contributing to the acid stability of GH10 xylanases, including electrostatic repulsion, π-π stacking, ionic bonds, hydrogen bonds, and Van der Waals interactions. Furthermore, through mutagenesis studies, we uncovered that acid stability is influenced by a complex interplay of amino acid residues. The key amino acid sites determining the acid stability of GH10 xylanases were thus elucidated, mainly concentrated in two surface regions behind the enzyme active center. Notably, the critical residues associated with acid stability markedly enhanced Xyn10RE’s thermostability by more than sixfold, indicating a potential acid-thermal interplay in GH10 xylanases. This study not only reported a series of valuable genes but also provided a range of modification targets for enhancing the acid stability of GH10 xylanases.

**Key points:**

*• Five acid stable and thermostable GH10 xylanases were reported.*

*• The key amino acid sites, mainly forming two enriched surface regions behind the enzyme active center, were identified responsible for acid stability of GH10 xylanases.*

*• The finding revealed interactive amino acid sites, offering a pathway for synergistic enhancement of both acid stability and thermostability in GH10 xylanase modifications.*

**Supplementary Information:**

The online version contains supplementary material available at 10.1007/s00253-024-13045-1.

## Introduction

Xylanases, as glycoside hydrolases, catalyze the hydrolysis of internal β-1,4 bonds in xylan (Collins et al. 2005; Dodd and Cann 2009), the second most abundant polysaccharide on earth. Secreted by microbes like bacteria and fungi, these enzymes with different properties degrade plant biomass into soluble sugars, facilitating microbial growth (Elkins et al. 2010; Chaudhary et al. 2021). With wide-ranging applications, microbial xylanases serve critical roles across diverse industries. In the area of biofuels, for instance, they are crucial catalysts for hydrolyzing polysaccharides within straw, leading to the production of oligosaccharides (Dodd and Cann 2009). In pulp processes, they aid in bleaching, effectively reducing chlorine usage (Sharma et al. 2020). In the animal feeding, they digest arabinoxylans in grains, thus enhancing feed utilization (Bajaj and Mahajan 2019). In addition, xylanases are also employed in food, beverage, and textile industries for various processes (Shahrestani et al. 2016; Yegin 2017; Abd El Aty et al. 2018). Recent research is channeling efforts into uncovering novel xylanases and refining the existing ones, with a keen focus on stability and activity under high temperatures and extreme pH levels, aiming to meet evolving industrial demands.

According to the CAZy database, xylanases are mainly categorized into glycoside hydrolase (GH) families 10 and 11 (Drula et al. 2022). GH10 xylanases exhibit a (α/β)8-barrel fold structure and weigh approximately 35 kDa (Collins et al. 2005), whereas GH11 xylanases, weighing around 20 kDa, are structured as a β-sandwich composed of α-helix and two β-sheets (Collins et al. 2005). While GH11 xylanases show specificity in recognizing xylan substrates (Paës et al. 2012), GH10 xylanases demonstrate greater catalytic versatility, capable of degrading highly branched xylan backbones, and even directly hydrolyzing cellulosic substrate (Chu et al. 2017; Fredriksen et al. 2019; Wang et al. 2019). Given their specialized role as “true xylanase,” GH11 xylanases have been extensively studied under extreme conditions, with acidophilic variants reported in various microbes, including *Acidobacterium*, *Trichoderma*, *Penicillium*, and *Aspergillus* (Collins et al. 2005; Paës et al. 2012). The adaptation of GH11 xylanases to low pH is hypothesized to arise from an increase in surface negative charge, mitigating electrostatic repulsion of positive residues at low pH (Li et al. 2021b). Alkali-stable enzymes, conversely, tend to exhibit fewer acidic residues and more arginines (Bai et al. 2015). As well, hydrophobicity, π-π stacking, and salt bridges also contribute to the acid stability of GH11 xylanases (de Lemos et al. 2004; Bai et al. 2015; Ge et al. 2019).

Nevertheless, the mechanisms driving pH stability in hydrolases, such as xylanases, are not yet fully understood. Despite the wealth of xylanase structures available in the Protein Data Bank (PDB) database, we still lack a thorough understanding of the structural basis for their pH stability, a property influenced by numerous factors. Beyond this, as research into xylanase applications continues to deepen, our scope of study should extend beyond just the GH11 family. For instance, GH10 xylanases have been noted for their superior performance in synergistic plant biomass deconstruction (Hu and Saddler 2018). Moreover, employing acidic enzymes for direct hydrolysis of acid-pretreated straw can significantly enhance cost efficiency. However, there is a distinct lack of studies exploring the acid stability of these GH10 xylanases.

In this study, we initially investigated Xyn10C, a GH10 xylanase from *Aspergillus fumigatus* Z5, exhibiting both thermal and acid stability. This led to the identification of a series of thermostable and/or acid stable GH10 xylanases. Subsequent analysis evaluated various factors affecting GH10 xylanases’ acid stability, including electrostatic repulsion, π-π stacking, ionic bonds, hydrogen bonds, and Van der Waals interactions. Further insights into the acid stability of GH10 xylanases were gained through comparing protein structures and conducting mutational analyses, identifying key amino acid residues in the process.

## Materials and methods

### Strains and culture conditions

*A. fumigatus* Z5 (CGMCC Accession No. 3309) was stored in our lab and used for the GH10 xylanase study. Fungal liquid cultivation was performed with 250-mL flasks by inoculating 1 × 10^7^ fresh conidia or 0.1 g fresh mycelia into 50 mL of Mandels’ salt solution (0.25 g·L^−1^ yeast extract, 1 g·L^−1^ MnSO_4_·H_2_O, 0.5 g·L^−1^ urea, 0.5 g·L^−1^ (NH_4_)_2_SO_4_, 0.5 g·L^−1^ CaCl_2_, 0.5 g·L^−1^ MgSO_4_·7H_2_O, 7.5 mg·L^−1^ FeSO_4_·7H_2_O, 2.5 mg·L^−1^ MnSO_4_·H_2_O, 3.6 mg·L^−1^ ZnSO_4_·7H_2_O, 3.7 mg·L^−1^ CoCl_2_·6H_2_O) supplemented with 2% (w/v) glucose or oat spelt xylan (Sigma-Aldrich, St. Louis, USA). Fungal cultures were incubated at 37 °C and 150 rpm. *Pichia pastoris* X33 (Invitrogen, Waltham, USA) was used as the host for protein heterologous expression. The YPDS medium (2% peptone, 1% yeast extract, 2% glucose, and 1 M sorbitol, pH 6.0) with 100 µg·mL^−1^ Zeocin (Sigma-Aldrich, St. Louis, USA) was used for transformant screening. BMGY/BMMY (2% peptone, 1% yeast extract, 1.34% YNB, 4 × 10^−5^% biotin, 1% glycerol, or 0.5% methanol, pH 6.0) was used as the growth/induction medium for enzyme production by *P. pastoris* X33. Plasmids were stored in *Escherichia coli* Top10 (Invitrogen, Waltham, USA) cultivated in the low-salt Luria–Bertani medium (1% peptone, 0.5% yeast extract, 0.5% NaCl, pH 7.0).

### Heterologous protein expression and purification

The mycelia of *A. fumigatus* Z5 was first induced by 1% (w/v) xylan for 10 h and then used to extract total RNA using RNeasy Plant Mini Kit (Qiagen, Hilden, Germany). A 1-µg mass of total RNA with good quality was then used as the template for cDNA synthesis using PrimeScript™ RT-PCR Kit (Takara, Dalian, China). The *xyn10a* (Y699_04481), *xyn10b* (Y699_06333), and *xyn10c* (Y699_09486) genes were amplified from the above xylan-induced cDNA. For Xyn10C mutants and other GH10 xylanases, such as Xyn10AR (EYE97139.1), Xyn10AO (AFP43760.1), Xyn10PR (CDM37719.1), Xyn10TC (BAN82655.1), Xyn10BS (ACS96449.1), Xyn10PC (ACP27611.1), Xyn10RE (CAD34597.1), and its mutants, their gene sequences were synthesized directly by the company (Tsingke, Beijing, China) and verified by DNA sequencing. The signal peptide was predicted using SignalP 5.0 software (Almagro Armenteros et al. 2019).

These GH10 xylanase genes were amplified and inserted into pPICZαA through the multiple cloning sites to form each expression vector, which was linearized with PmeI enzyme (New England Biolabs, Beijing, China) and then transformed into *P. pastoris* X33 followed by the screening on YPDS plates containing 100 µg·mL^−1^ of Zeocin. The correct mutants were cultured in 50 mL BMGY medium in a 250-mL flask for 20 h at 30 °C and 200 rpm, and then, the cells were transferred into the fresh BMMY medium for enzyme induction. Every 24 h, 100% methanol was added to a final concentration of 1% in the medium. Crude enzymes were prepared using the ammonium sulfate precipitation, and enzyme purification was carried out using a Sephadex G-200 column (GE Healthcare, NJ, USA) as described previously (Miao et al. 2015). The correct transformant was confirmed by its extracellular xylanase activity and SDS-PAGE.

### Enzyme activity assay

The substrate solution for xylanase assays consisted of 1% (w/v) oat spelt xylan (Sigma-Aldrich, St. Louis, USA) in a sodium acetate buffer (50 mM, pH 4–6) with the optimal pH. A total of < 5 µL of purified xylanase was mixed with 1 mL of substrate solution and incubated for 10 min at the optimal temperature. Then, the reaction was terminated by adding 1 mL of 3,5-dinitrosalicylic acid (DNS) and boiled for 10 min. The concentration of released reducing sugars was determined by measuring the absorbance at 520 nm with the standard of d-xylose (Sigma-Aldrich, St. Louis, USA). One unit of enzyme activity was defined as the amount of enzyme required to release 1 µmol of reducing sugars from the substrate in 1 min.

For the optimal temperature, the reaction was detected at different temperatures ranging from 20 to 100 °C at the optimal pH. The thermostabilities were determined by incubating enzymes at different temperatures for 6 h at the optimal pH, and then, the residual xylanase activities were measured in each sampling time (10 min, 30 min, 1 h, 2 h, 4 h, and 6 h) at the optimal temperature and pH. For the optimal pH, different pH values in substrate solution were prepared using 50 mM HCl-KCl buffer (pH 1.0–2.0), 50 mM citrate buffer (pH 3.0–6.0), 50 mM PBS buffer (phosphate-buffered saline, pH 6.0–8.0), and 50 mM glycine–NaOH buffer (pH 8.0–11.0). pH stabilities were determined by incubating each enzyme in the different pH buffers for 1 h at 4 °C followed by activity determination at the optimal temperature and pH.

The protein concentration was estimated with a Micro BCA protein assay kit (Beyotime, Shanghai, China), and then, the specific activity towards the oat spelt xylan was determined at the optimal conditions. The *K*_m_ constant was determined by incubating (10 min) a fixed amount of xylanase with varied concentrations of oat spelt xylan (2.5 to 15 mg·mL^−1^) at the optimal temperature and pH. The data obtained were fitted to the standard Michaelis–Menten model.

### SDS-PAGE analysis

The protein concentration was determined using a Micro BCA protein assay kit (Beyotime, Shanghai, China). Xyn10C deglycosylation was performed using Endo-H enzyme (New England Biolabs, Beijing, China) with or without preheating at 100 °C for 10 min before hydrolyzing. Sodium dodecyl sulfate–polyacrylamide gel electrophoresis (SDS-PAGE) was performed on a 10% (w/v) polyacrylamide gel with a protein marker of PageRuler Prestained Protein Ladder (Fermentas, Shenzhen, China) using a Mini-PROTEAN Tetra Cell Systems (Bio-Rad, Hercules, USA). The target proteins on the gel were visualized by straining with Coomassie Brilliant Blue R-250 according to the manufacturer’s instructions.

### Phylogenetic analysis of GH10 xylanases

The protein sequences of Xyn10C and Xyn10A were used to search the Universal Protein Resource Knowledgebase (UniProtKB) database (https://www.uniprot.org), and the matched proteins with identity > 60% were downloaded and combined uniquely. Protein alignment was performed with ClustalX 2.1 (http://www.clustal.org, Larkin et al. 2007) and manually processed using AliView 1.23 (https://ormbunkar.se/aliview/, Larsson 2014). The resulting matrix was subjected to Bayesian analysis using MrBayes v3.2.6 (https://nbisweden.github.io/MrBayes/, Ronquist et al. 2012). The best-fit model of protein evolution was WAG + I + G + F identified by ProtTest 3.2 (https://github.com/ddarriba/prottest3, Darriba et al. 2011). Two simultaneous, completely independent analyses starting from different random trees were run for 5 million generations, each with four chains, with a sampling frequency of every 1000 and a burn-in of 25%. Bayesian posterior probability (PP) values lower than 0.95 were not considered significant, while values below 0.9 were not shown on phylograms.

### Homology modeling and protein comparison of GH10 xylanases

The 3D structures of GH10 xylanases were constructed using the SWISS MODEL server (http://swissmodel.expasy.org) (Waterhouse et al. 2018). The models were then viewed and analyzed using PyMOL v2.0 software (https://pymol.org/2/). To ensure the accuracy of comparison, the only reference protein of GH10 xylanase was selected to be Xyn10A, which has a precise crystalline structure (PDB ID: 6JDT) reported by our previous study (Li et al. 2021a, b). Different acidic/basic amino acid residues around/in the enzyme active center between Xyn10C and Xyn10A were selected directly as the targets. Structure superposition in PyMOL was used to assist in protein comparison for identifying the exact location of different residues. In addition, all acid stable GH10 xylanases in cluster IV and non-acid stable Xyn10A were aligned at protein level using ClustalX 2.1 to detect the different amino acid residues. Xyn10RE, the closest acid stable xylanase to Xyn10A in the phylogenetic tree, was further used as the template for the subsequent substitution design.

### Statistical data analysis

The residue interaction networks included six interactions of hydrogen bonds, Van der Waals, disulfide bonds, ionic bonds, π-π stacking, and π-cation with the cutoff distances of corresponding atoms of 3.5 Å, 0.5 Å, 2.5 Å, 4.0 Å, 6.5 Å, and 5.0 Å, respectively (Clementel et al. 2022). The residue interactions were generated and counted at the RING server (http://ring.biocomputingup.it). The difference of acidic/basic residues and the residue interactions between different clusters of GH10 xylanases were evaluated by the *t*-test.

## Results

### Thermo and acid stability analysis of phylogenetically different GH10 xylanases in filamentous fungi

**Fig. 1:**
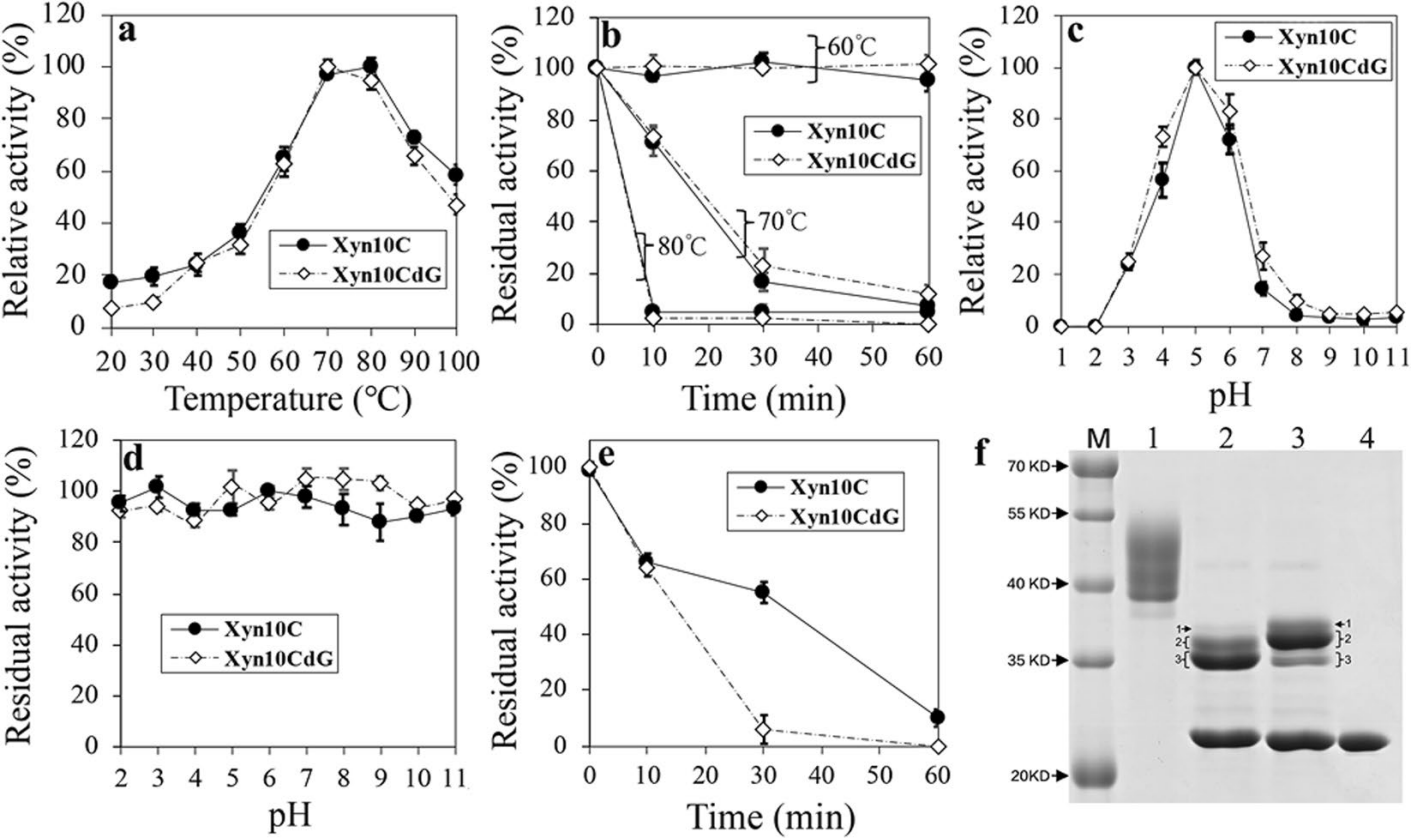
Enzymatic analysis of GH10 Xyn10C and the deglycosylated Xyn10CdG. For Xyn10C and Xyn10CdG, their enzymatic properties, such as the optimal temperature (**a**), thermostability (**b**), the optimal pH (**c**), and pH stability (**d**), were detected and shown. In pH stability, the residual activities of Xyn10C and Xyn10CdG at pH 3.0 to 11.0 were results detected after the incubation of 1 h at 4 °C; however, those at pH 2.0 were the results of 6-h incubation. The stabilities of Xyn10C and Xyn10CdG at pH 1.0 were determined and showed separately (**e**). SDS-PAGE (**f**) showed the purified Xyn10C (line 1), the deglycosylated Xyn10CdG treated by the heating at 100 °C for 10 min before the Endo H hydrolyzation (line 2), the deglycosylated Xyn10CdG directly hydrolyzed by Endo H (line 3), and single Endo H enzyme (line 4)

Xyn10C (accession no.: Y699_09486), a GH10 xylanase from *A. fumigatus* Z5, showed optimal activity at 80 °C and a pH of 5.0 (Fig. 1a–c). Unlike most GH10 xylanases, which rapidly lost activity below pH 3.0 at 4 °C, Xyn10C maintained its highest xylanase activity even after 6 h of incubation at pH 2.0 at 4 °C (Fig. 1d). Remarkably, it retained over 50% of activity after 30 min of incubation at pH 1.0 at 4 °C, highlighting its high thermostability and acid stability (Fig. 1e). With a specific activity of 183.62 U·mg^−1^ and a *K*_m_ value of 12.04 mg·mL^−1^ for 1% (w/v) oat spelt xylan, Xyn10C appeared heavily glycosylated according to SDS-PAGE result (Fig. 1f). However, while glycosylation was observed to interfere with Xyn10C’s substrate affinity (the deglycosylated *K*_m_ value of 9.93 mg·mL^−1^, *p* < 0.001), it did not significantly affect its thermostability and acid stability (Fig. 1b and d).

**Fig. 2:**
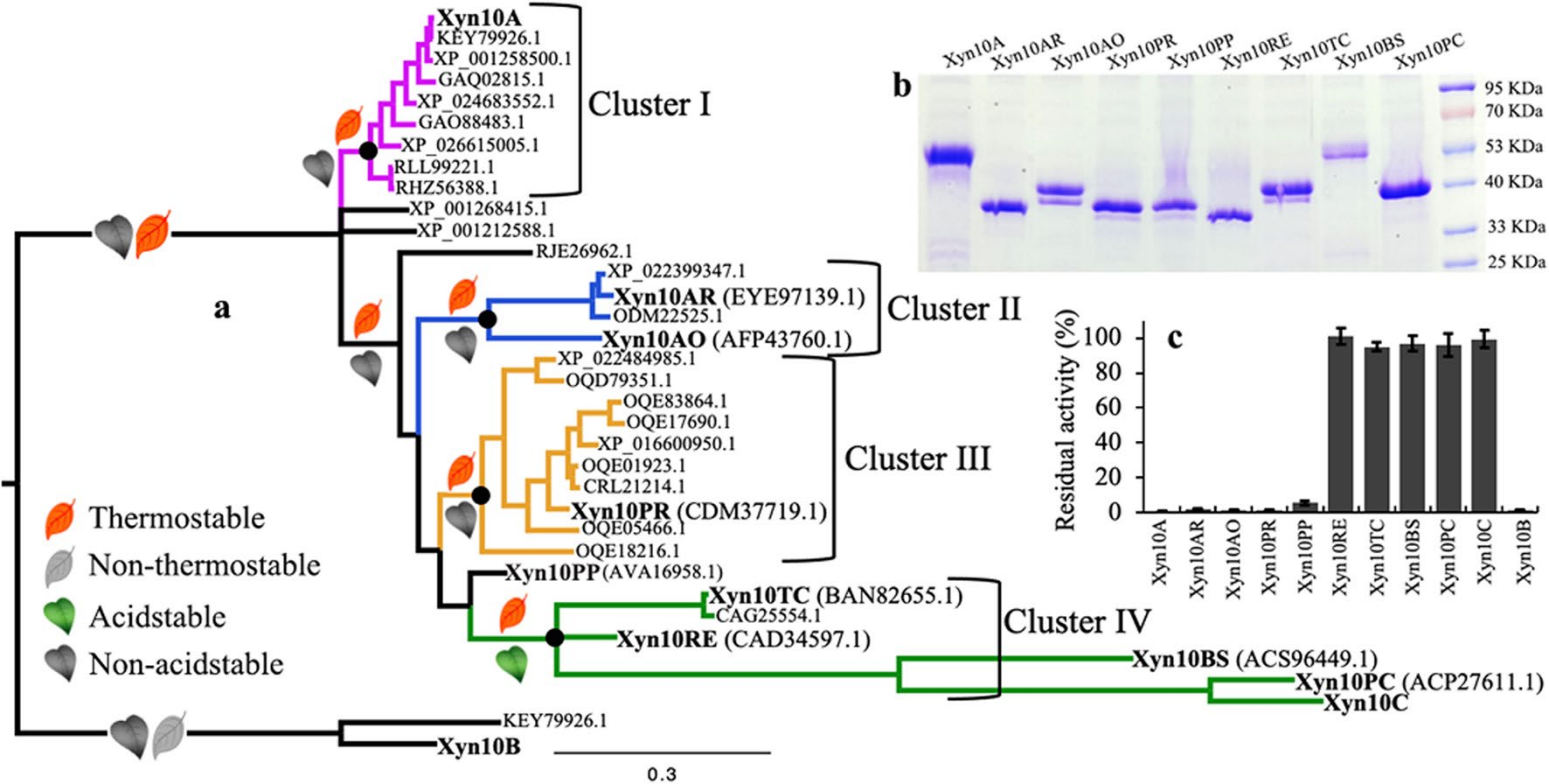
Phylogenetic analysis of Xyn10C-related GH10 xylanases in filamentous fungi. The phylogenetic tree of GH10 xylanases, constructed from UniProtKB database using Xyn10A (Y699_04481) and Xyn10C (Y699_09486) as templates, resulted in four distinct evolutionary clusters (I in pink, II in blue, III in brown, IV in green) (**a**). Each cluster’s most recent common ancestor points were marked by black solid circles, enabling mutant rate analysis within these clusters. Key proteins, including Xyn10A in cluster I (pink); Xyn10AR and Xyn10AO in cluster II (blue); Xyn10PR in cluster III (brown); Xyn10PP, Xyn10TC, Xyn10RE, Xyn10BS, and Xyn10PC in cluster IV (green), were heterologously expressed and purified (**b**). These proteins were then tested for acid stability by incubating at pH 2.0 for 1 h at 4 °C and then detecting the residual activities (**c**)

To further explore the determinants of acid stability, a phylogenetic tree of related GH10 xylanases was constructed using Xyn10C as a reference in the UniProtKB database. The tree divided into four distinct clusters, with cluster IV notably evolving at an accelerated rate (Fig. 2a). Subsequently, a range of GH10 xylanases was heterologously expressed, including Xyn10A from cluster I; Xyn10AR and Xyn10AO from cluster II; Xyn10PR from cluster III; and Xyn10PP, Xyn10TC, Xyn10RE, Xyn10BS, and Xyn10PC from cluster IV (Fig. 2b). Due to the rapid inactivation of most GH10 xylanases at pH 2.0 and its applicability, this pH was selected for efficiently discriminating the acid stability of GH10 xylanases. GH10 xylanases from clusters I, II, and III (Xyn10A, Xyn10AR, Xyn10AO, and Xyn10PR) proved thermostable with optimal reaction temperatures between 70 and 90 °C (Supplemental Table S1), but lacked stability at pH 2.0 (Fig. 2c). Xyn10PP, situated close to the root of cluster IV, did not survive the acidic condition of pH 2.0. Conversely, Xyn10TC, Xyn10RE, Xyn10BS, and Xyn10PC from cluster IV were all stable at pH 2.0 (Fig. 2c) and also displayed thermostability (Supplemental Table S1). These results indicate that clusters I, II, and III harbor highly thermostable GH10 xylanases, whereas cluster IV has adapted to exhibit acid stability. Additionally, the persistent acid stability and high mutation rates of GH10 xylanases in cluster IV, as indicated by an average branch length of 0.57 compared to 0.07, 0.17, and 0.14 in clusters I, II, and III, respectively (Fig. 2a, p < 0.001), implied significant selective pressure. Therefore, these results elucidate the evolutionary relationship between thermostability and acid stability in GH10 xylanases from filamentous fungi and delineate a unique branch, cluster IV, that exhibits both qualities.

**Fig. 3:**
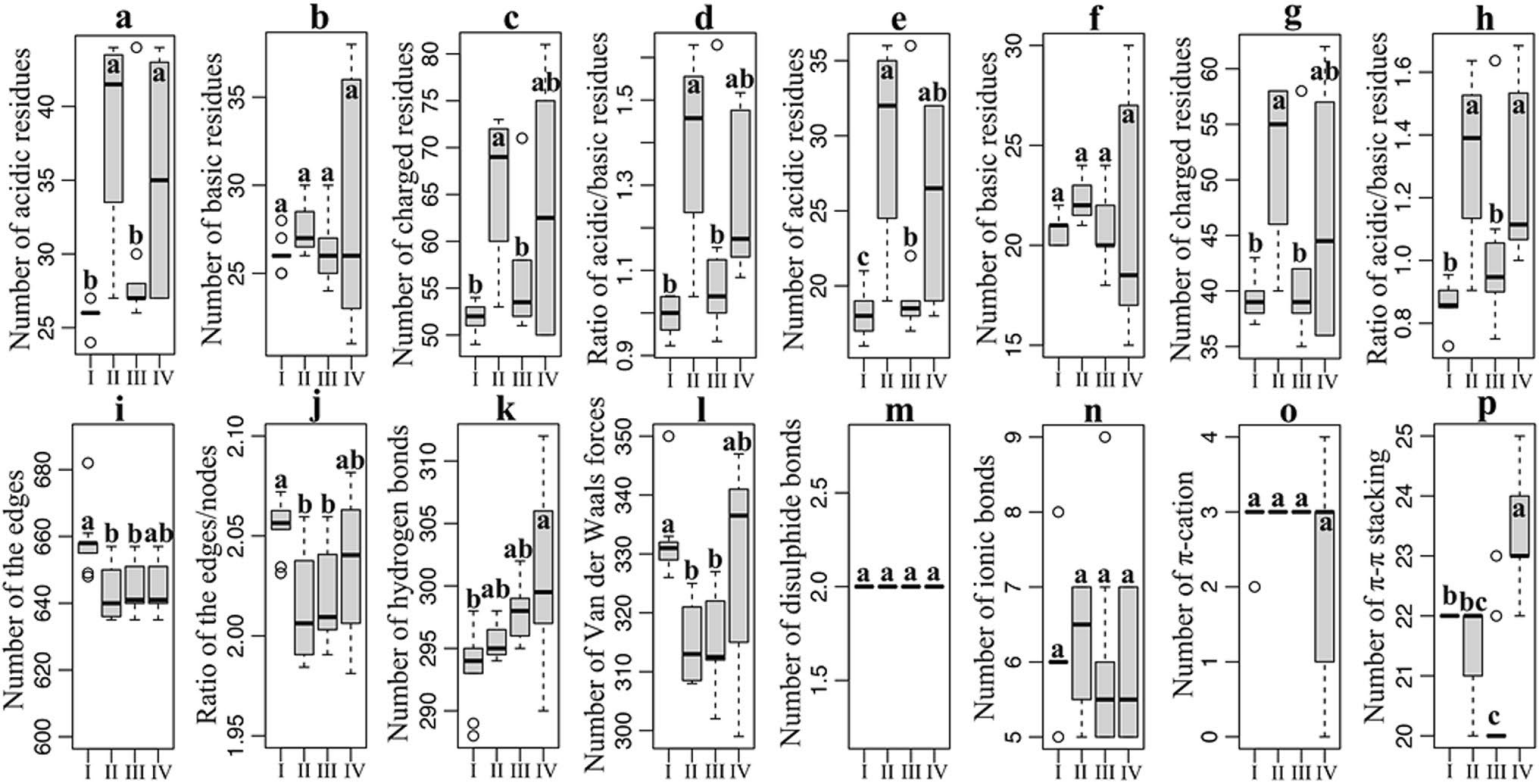
Statistical analysis of factors possibly affecting the low pH tolerance of GH10 xylanases. The distribution of the charged amino acids in the whole protein (**a**–**d**) or just on the protein surface (**e–h**), including acidic residues of D and E, as well as basic residues of R, K and H, was counted and compared between different clusters (I, II, III, and IV) in the Xyn10C-derived phylogenetic tree. Factors such as the number of acidic residues, the number of basic residues, the number of charged residues, and the ratio of acidic to basic residues were examined. Meanwhile, non-covalent interactions, such as total number of interactions (edges) (**i**), ratio of interactions to residues (nodes) involved (**j**), hydrogen bonds (**k**), Van der Waals forces (**l**), disulfide bonds (**m**), ionic bonds (**n**), π-cation (**o**), and π-π stacking (**p**), were also compared. Different letters on boxes denote statistically significant differences as assessed by the *F*-test followed by the *t*-test (*p* < 0.05)

A detailed comparison of acidic and basic residues between acid stable and non-acid stable GH10 xylanases, using the Xyn10C-derived phylogenetic tree, found cluster IV demonstrating a higher count and ratio of acidic residues, both overall and on the protein surface (Fig. 3a–h). This may suggest a common adaptive strategy under low pH conditions, possibly mitigating the structural damage from electrostatic repulsion. Yet, the similar acidic residue counts in clusters IV and II (Fig. 3a–h) indicate other contributing factors. Acid stability, influenced by intricate residue interactions with 3D structure, was thus explored in terms of non-covalent interactions. No significant difference was observed in edges and edge-to-node ratio across clusters IV, I, II, and III (Fig. 3i and j), indicating comparable interaction counts and averages per amino acid among acid stable and non-acid stable GH10 xylanases. Additionally, there were also no notable differences across specific interaction types, such as hydrogen bonds, Van der Waals forces, disulfide bonds, ionic bonds, and π-cation interactions (Fig. 3k–o), thus reducing their apparent significance. Conversely, π-π stacking interactions between aromatic residues, particularly Phe-Phe (F-F) and Phe-Tyr (F-Y), were significantly more frequent in cluster IV (Fig. 3p). Particularly noteworthy was the F-F interaction which, while almost non-existent in clusters I, II, and III, was prevalent in acid stable GH10 xylanases of cluster IV with a mean of 3.2 ± 2.9 (Supplemental Table S2). These data emphasize the pivotal role of acidic residues and π-π stacking in enhancing GH10 xylanases with tolerance to low pH conditions.

### Multiple sites far from the active center jointly determine the acid stability of GH10 xylanases

Given the apparent link between acid stability and acidic/basic residues on the protein surface, we first explored residues near Xyn10C’s active center. We compared acid stable Xyn10C to non-acid stable Xyn10A, identifying five residues in Xyn10C (A113, S116, G161, E244, and E326) with acidic/basic amino acid differences. After modifying these residues to match Xyn10A, we obtained the mutants Xyn10C_A113H-S116R, Xyn10C_G161E, Xyn10C_E244S, and Xyn10C_E326Q (Supplemental Fig. S1a). However, these mutants displayed no significant changes in acid stability compared to Xyn10C (Supplemental Fig. S1b). Turning to the acidic/basic amino acids within the enzyme active center, we found an E237 in Xyn10C replaced by Q in Xyn10A. While the mutant Xyn10C_E237Q retained similar xylanase activity, Xyn10C_E237G retained only ~ 5% activity, and Xyn10C_E237L completely lost xylanase activity. This suggests the presence of E or Q at this site is vital for enzyme function. Although the E/Q site mechanism in GH10 xylanases requires further exploration, this site did not significantly impact Xyn10C’s acid stability (Supplemental Fig. S1b). Furthermore, E157 and E269 in Xyn10C, key catalytic residues for GH10 xylanases, were rendered completely inactive when mutated (Xyn10C_E157Q and Xyn10C_E269Q). Thus, we can conclude that the acidic/basic amino acids in and around the enzyme active center are not responsible for the low pH tolerance of GH10 xylanases.

**Fig. 4:**
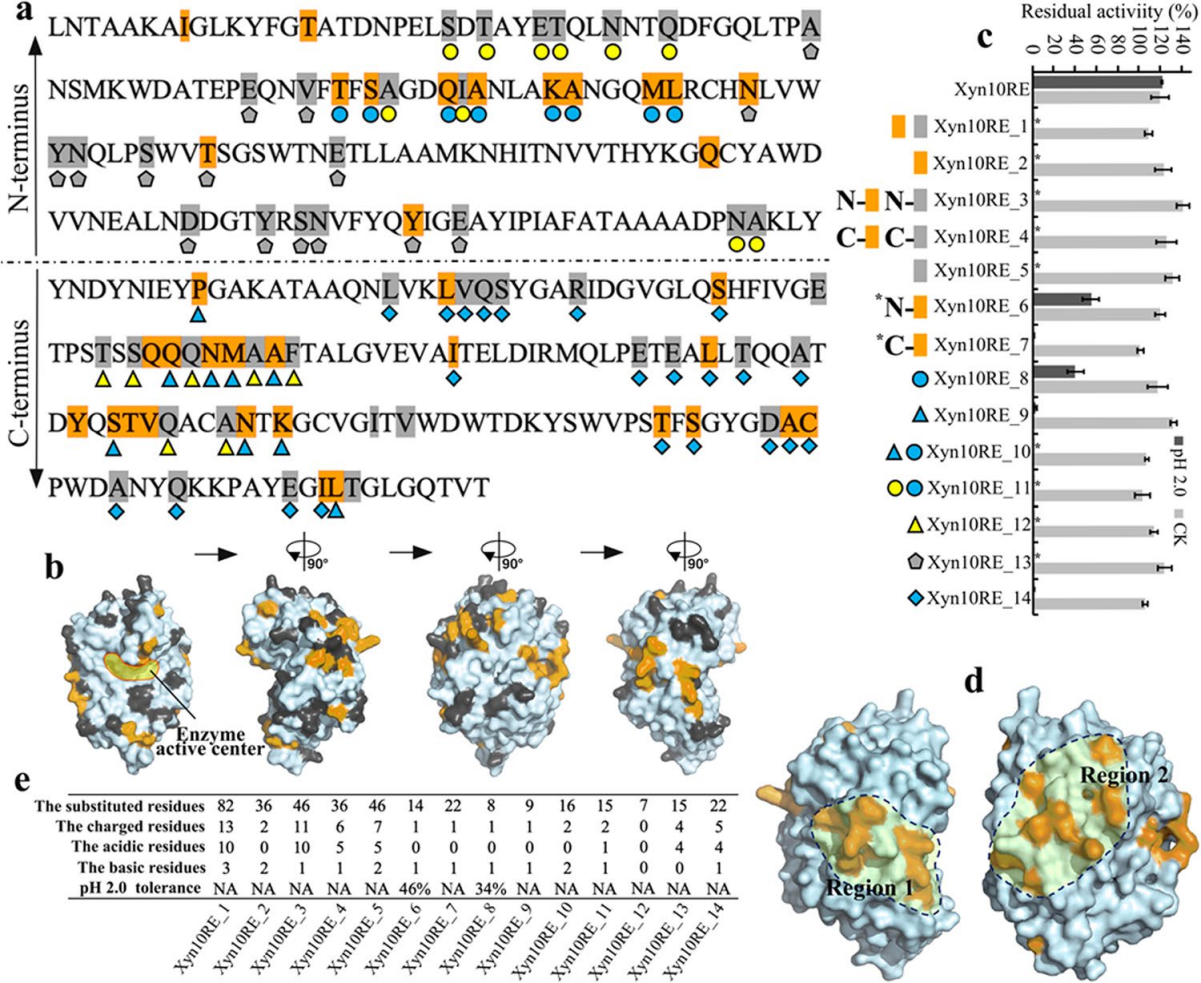
Screening and identification of key amino acid residues affecting the low pH tolerance of GH10 xylanase, Xyn10RE. A protein sequence of Xyn10RE is shown in (**a**), where the brown positions represent group A amino acid residues that differ in that position from Xyn10A in all GH10 xylanases of cluster IV, while the gray positions represent group B amino acid residues that differ in that position from Xyn10A in only part GH10 xylanases of cluster IV. Mutations at specific amino acid sites are marked with different colored shapes (blue, yellow, and gray) such as circles, triangles, diamonds, and pentagons. These different amino acid residues are also highlighted on the Xyn10RE’s simulated 3D structure (**b**), with the enzyme active center indicated by a yellow shadow. Fourteen Xyn10RE mutants were constructed by substituting various group A or B residues, indicated by adjacent different colors or color-labeled shapes. Their acid stabilities were assessed by measuring residual activities after 1 h at pH 2.0 for each enzyme (**c**). CK refers to xylanase activities without pH 2.0 treatment of Xyn10RE and its mutants. On the Xyn10RE protein structure, two enriched regions (1 and 2) of the group A amino acid residues are indicated and circled (**d**). Acidic/basic residues distributed in different Xyn10RE mutants are listed (**e**). *N/C-tagged colors mean that color-labeled residues at N/C-terminus

In light of the complex acid stability mechanism, we ceased studying the effects of other acidic/basic residues on Xyn10C. Instead, we selected the acid stable Xyn10RE from acid stable cluster IV, which is most closely related to non-acid stable Xyn10A, for a comprehensive sequence difference analysis with Xyn10A. This focused approach aimed to capture more factors influencing acid stability. Our analysis identified 82 amino acid variances in Xyn10RE relative to Xyn10A. These variances were categorized into two groups: group A (Fig. 4a and b, brown), comprising residues where all cluster IV GH10 xylanases (5/5) also differed from Xyn10A and group B (Fig. 4a and b, gray), including residues where only a subset (≤ 4/5) of cluster IV GH10 xylanases showed differences from Xyn10A. Four Xyn10RE mutants were first constructed: Xyn10RE_1 had all differing amino acids replaced with Xyn10A residues, Xyn10RE_2 had all group A residues replaced, Xyn10RE_3 and Xyn10RE_4 had half of group A and B residues replaced at the N and C-termini, respectively (Fig. 4a, Supplemental Table S3). Acid stability tests revealed that all mutants lost all xylanase activity after 1-h incubation at pH 2.0 (Fig. 4c), suggesting these differing amino acids significantly contribute to Xyn10RE’s low pH tolerance. The total loss of activity in Xyn10RE_2, Xyn10RE_3, and Xyn10RE_4 mutants (Fig. 4c) further underscore the decisive role of group A amino acids in acid stability, while not dismissing potential contributions from group B. Subsequent mutants, Xyn10RE_5 (all group B residues replaced), Xyn10RE_6 (half group A replaced at N-terminus), and Xyn10RE_7 (half group A replaced at C-terminus) were also tested (Fig. 4a, Supplemental Table S3). Xyn10RE_5 lost all acid stability at pH 2.0, indicating the importance of group B. Xyn10RE_6 retained ~ 46% of xylanase activity after 1 h at pH 2.0, suggesting that residues in groups A and B at the N-terminus collectively influence low pH tolerance. Meanwhile, Xyn10RE_7 completely lost its acid stability, highlighting the critical role of group A residues at the C-terminus. These results highlight the importance of amino acids in groups A and B. Yet, to identify the specific residues essential for Xyn10RE’s tolerance to low pH, a more focused investigation is necessitated.

The 3D structure of Xyn10RE was thus simulated to highlight the differential residues between Xyn10RE and Xyn10A (Fig. 4b). Two regions (1 and 2), rich in group A residues, were identified (Fig. 4d). Xyn10RE’s group A residues from these regions were located at the N-terminus and the C-terminus, respectively, in the primary structure. Mutants of Xyn10RE_8, Xyn10RE_9 and Xyn10RE_10, which included substitutions in region 1, region 2, and both, respectively, were constructed (Fig. 4a, Supplemental Table S3). Following 1-h incubation at pH 2.0, Xyn10RE_8, like Xyn10RE_6, retained approximately 34% of xylanase activity (Fig. 4c); however, the number of amino acid substitutions in Xyn10RE_8 decreased further from 14 to 8. Xyn10RE_9, included in Xyn10RE_7, completely lost its acid stability at pH 2.0 (Fig. 4c), but only nine residues substituted. Xyn10RE_10, which covered both Xyn10RE_8 and Xyn10RE_9, lost 100% of acid stability (Fig. 4c). This suggests that Xyn10RE’s acid stability is likely governed by many residues, primarily distributed in and around regions 1 and 2. To validate this, we constructed four additional mutants: Xyn10RE_11 (including Xyn10RE_8 plus group B residues in region 1), Xyn10RE_12 (substituting group B residues in region 2), Xyn10RE_13 (substituting groups A and B residues outside region 1 at the N-terminus), and Xyn10RE_14 (substituting groups A and B residues outside region 2 at the C-terminus) (Fig. 4a, Supplemental Table S3). Upon exposure to pH 2.0, Xyn10RE_11 entirely lost its acid stability (Fig. 4c), reinforcing that the synergistic action of groups A and B residues in region 1 is crucial for Xyn10RE’s low pH tolerance. Similarly, the group B residues in region 2 played a significant role in Xyn10RE_12 losing its acid stability at pH 2.0 (Fig. 4c). These findings strongly demonstrate the importance of regions 1 and 2 in contributing to the low pH tolerance of Xyn10RE. Results of Xyn10RE_13 and Xyn10RE_14 (Fig. 4c) further reflected the complexity of the acid stability mechanism, implying residues outside regions 1 and 2 could still drastically influence Xyn10RE’s acid stability. Importantly, these key residues influencing the acid stability of GH10 xylanase are conserved in cluster IV despite a relatively high mutation rate, pointing to a strong selective pressure for maintaining GH10 xylanase acid stability.

Based on the Xyn10RE mutants, we also analyzed the contribution of acidic/basic amino acids and the non-covalent interactions to the low pH tolerance of GH10 xylanases (Fig. 4e). As shown, acidic residues were relatively enriched in the different amino acids between Xyn10RE and Xyn10A. However, in mutants like Xyn10RE_2, Xyn10RE_6, Xyn10RE_7, Xyn10RE_8, Xyn10RE_9, Xyn10RE_10, and Xyn10RE_12, loss of low pH tolerance primarily resulted from substitutions not involving acidic residues. Moreover, despite its potential contribution to low pH tolerance, π-π stacking did not quantitatively change across different Xyn10RE mutants (Supplemental Table S4). Rather, fluctuations in hydrogen bond and Van der Waals interactions were observed among Xyn10RE mutants (Supplemental Table S4). Rather, fluctuations in hydrogen bond and Van der Waals interactions were observed among Xyn10RE mutants (Supplemental Table S4). These findings suggest that acid stability of GH10 xylanases is a complex structural issue, involving numerous contributing factors.

### Amino acid residues affecting acid stability also regulate thermostability

**Fig. 5:**
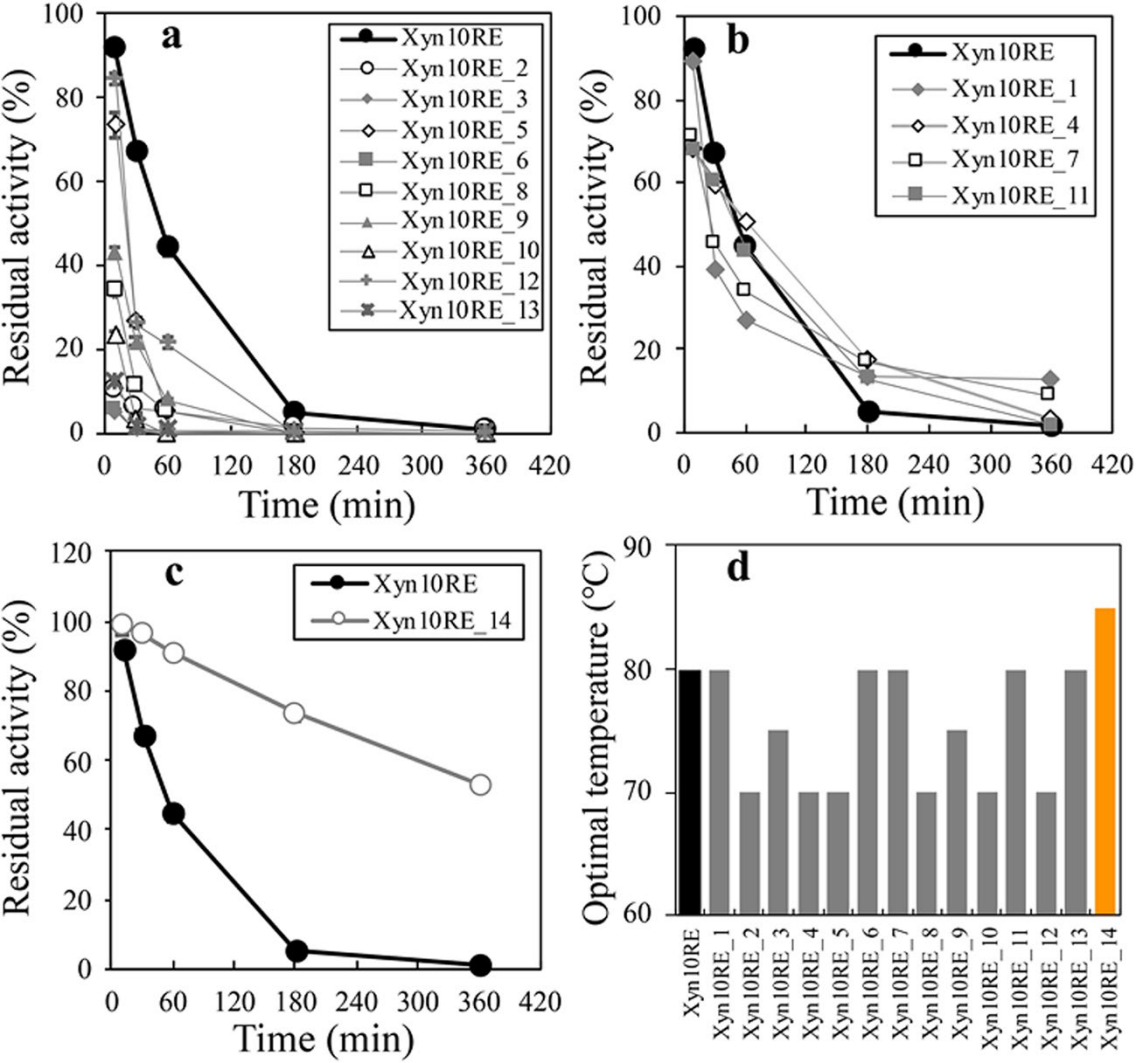
The optimal temperature and thermostability of Xyn10RE and its mutants. After the amino acid substitutions, Xyn10RE was reconstructed into the 14 different mutants (Xyn10RE_1 to Xyn10RE_14). In order to assess their thermostabilities, the residual activities were measured at each optimal temperatures and pHs after incubation at 70 °C for 10 min, 30 min, 1 h, 3 h, and 6 h, respectively. Mutations in these enzymes resulted in a dramatic reduction in thermostability (**a**), a larger drop-off in residual activity than Xyn10RE at the early stage but a more sustained activity (**b**), and a largely improved thermostability (**c**). Their optimal temperatures were also detected and shown in (**d**)

Due to the coupled relationship of thermostability and acid stability in GH10 xylanases of cluster IV, we further detected the residual activities of different Xyn10RE mutants after incubation at a high temperature of 70 °C. The results showed a large variation in their thermostabilities. Specifically, seven mutants, namely, Xyn10RE_2, Xyn10RE_3, Xyn10RE_6, Xyn10RE_8, Xyn10RE_9, Xyn10RE_10, and Xyn10RE_13, showed drastically reduced or completely lost thermostability (Fig. 5a). Furthermore, Xyn10RE_1, Xyn10RE_4, Xyn10RE_5, Xyn10RE_7, Xyn10RE_11, and Xyn10RE_12 exhibited decreased thermostability. Notably, despite an initial rapid decrease within 30 min, Xyn10RE_1, Xyn10RE_4, Xyn10RE_7, and Xyn10RE_11 maintained a significantly longer enzymatic activity duration compared to Xyn10RE (Fig. 5b). Also, thermostability showed a complex association with residue substitution. For instance, complete substitution in Xyn10RE_1 had a minimal impact on thermostability, whereas partial substitution in Xyn10RE_2 or Xyn10RE_3 resulted in a complete loss of thermostability.

Surprisingly, Xyn10RE_14 showed a significant improvement in thermostability when compared to the original Xyn10RE. Approximately 5% of the residual activity of Xyn10RE after 3 h at 70 °C was largely increased to maintain more than 50% of the residual activity of Xyn10RE_14 after 6 h at 70 °C (Fig. 5c). The optimal temperature of Xyn10RE_14 was increased to 85 °C (Fig. 5d). Noticeably, Xyn10RE_14 will be of value in applications with high demands for thermostability.

## Discussion

Xylanases facilitate plant biomass degradation by hydrolyzing the polysaccharide xylan, advancing their utility in industries like pulp bleaching, animal feeding, bioethanol, and baking (Collins et al. 2005). There is an increasing demand for xylanases with high activity and stability under severe conditions, like high temperatures and extreme pHs, to optimize industrial processes, enhance efficiency, and reduce microbial contamination. Filamentous fungi secrete various enzymes, including GH10 and GH11 xylanases, to synergistically degrade plant biomass (Gupta et al. 2016), and also exist as the main microbes under extreme environments (Hassan et al. 2019). Thus, they are a natural resource pool of valuable genes. From this aspect, Xyn10C, a GH10 endo-β-1,4-xylanase from *A. fumigatus* Z5, demonstrated high thermostability (optimal temperature 80 °C) and acid stability, retaining full activity at pH 2.0 for 6 h, unlike Xyn10A and Xyn10B from the same organism which were inactivated within 10 min at pH 2.0 (Miao et al. 2015). These traits permit Xyn10C’s application in acidic conditions, like animal feed and baking (Sharma et al. 2016). Unlike the GH11 family xylanases, acid stable GH10 xylanases have been less reported. Thus, based on the Xyn10C-derived phylogenetic tree, a series of GH10 xylanases were found to be thermostable, and the proteins in cluster IV, such as Xyn10RE, Xyn10TC, Xyn10BS, and Xyn10PC, were all both thermostable and acid stable. Intriguingly, acid stability and thermostability in GH10 xylanases are interlinked, with changes in one influencing the other in mutants like Xyn10RE. The resulted Xyn10RE_14 emerged as an exceptional mutant with superior thermostability, outperforming our previous deliberate modification of another thermostable GH10 xylanase, Xyn10A, achieved through rational design and saturation mutation. Xyn10AF-AC, the modified variant, improved Xyn10A’s residual activity by 8% to 45% after 4 h at 70 °C (Li et al. 2021a, b), while Xyn10RE_14 exhibited a significant leap, maintaining over 50% of its initial activity after 6 h at 70 °C, compared to the 5% residual activity of Xyn10RE after 3 h. Therefore, enzymes like Xyn10RE_14 also hold considerable industrial potential in extreme conditions, such as high temperatures and low pH levels.

In addition, the acid stability mechanism of GH10 xylanases was analyzed in depth in this study. Of course, there have been occasional reports on this aspect. For instance, GH10 XYL10C from *Bispora* sp. MEY-1 maintained > 90% of its activity at pH 1.5 for 1 h (Luo et al. 2009). GH10 ScXynA from *Scytalidium candidum* 3C displayed impressive acid tolerance, with a pH optimum of 3.5 (Eneyskaya et al. 2020). Nevertheless, acid stability research has primarily been concentrated on the GH11 family due to its strong specificity to xylan. Significant findings include the acid stable GH11 XynA (optimal pH of 2.0) from *Penicillium* sp. 40 and GH11 xylanase (optimal pH of 3.0) from *Penicillium glabrum* and the high concentration of acidic residues on the surface of GH11 XynC (optimal pH of 2.0) from *Aspergillus kawachii*, hypothesized to contribute to its pronounced acid stability (Fushinobu et al. 1998; Kimura et al. 2000; Knob et al. 2013). Decreasing basic residues enhances GH11 xylanase TlXynA’s acid tolerance, revealing the strategy of balancing acidic/basic residues on protein surfaces to maintain structure stability in acidic environments (Wu et al. 2020). This concept is further supported by the highly negatively charged surface of other acidophilic GHs like endoglucanase SSO1949 from *Sulfolobus solfataricus* and β-mannanase man5AP13 from *Phialophora* sp. P13 (Huang et al. 2005; Zhao et al. 2010). These studies highlight the strategy of modulating acidic and basic surface residues to maintain protein stability in acidic conditions. It is hypothesized that surface-charged residues affect enzymatic conformational stability through mechanisms like electrostatic interactions, hydrogen bonds, and interactions with surrounding water molecules (Warden et al. 2015; Pedersen et al. 2019). Therefore, this research initially focused on the distribution of acidic/basic residues in GH10 xylanases. Acid stable GH10 xylanases from cluster IV had significantly more acidic residues than clusters I and III. Moreover, between the acid stable Xyn10RE and the non-acid stable Xyn10A, acidic residues were enriched compared to basic residues. Substitution of acidic residues was observed in seven Xyn10RE mutants that completely lost acid stability, together supporting the notion that a higher count of acidic residues on the protein surface enhances low pH tolerance of GH10 xylanases. However, this electrostatic interaction-based strategy does not fully explain the complex acid stability of GH10 xylanases. The opposite case naturally exists, in which alkalophilic xylanases from family 11 have more negatively charged residues than nonalkalophilic xylanases (Bai et al. 2015). GH10 xylanases in cluster II, despite their high level of acidic residues, also lacked stability at pH 2.0. These results indicate the complexity of GH10 xylanase acid stability, which is probably a result balanced by different factors. As suggested, reduced hydrophobicity might be a crucial factor for acidophilic adaptation of GH11 xylanases (de Lemos et al. 2004). Certain acidophilic xylanases from the GH11 family exhibited more π-π stacking, which helped maintain stability in acidic environments (Ge et al. 2019). Acid stable GH10 xylanases in cluster IV also showed significantly higher counts of π-π stacking than those in clusters I, II, and III, emphasizing the importance of π-π stacking in non-covalent interactions. Moreover, the F-F and F-Y stacking types were significantly increased, a finding consistent with other enzymes like GH11 xylanases contributing to acid stability (Zhang et al. 2009; Ge et al. 2019). In contrast, various substituted residues in Xyn10RE mutants led to loss of acid stability, without changing the number of π-π stacking interactions. This outcome did not confirm the role of π-π stacking in acid stability of GH10 xylanases. Interestingly, π-π stacking has also been reported to enhance xylanase thermostability (Yang et al. 2015), and almost all GH10 xylanases in this study exhibited thermostability. Thus, π-π stacking may be an influencing factor for both the acid stability and thermostability of GH10 xylanases. Furthermore, different Xyn10RE mutants demonstrated variations in the number of hydrogen bonds, ionic bonds, and Van der Waals forces, suggesting their potential contributions to the low pH tolerance of GH10 xylanases. Given the various key residues influencing the acid stability of GH10 xylanases, it is posited that acid stability is associated with the overall structural rigidity of GH10 xylanases and likely stems from an interplay of these factors.

In conclusion, this study delineates the evolutionary relationship between the thermostability and acid stability of GH10 xylanases. It identifies several thermostable and acid stable GH10 xylanases, such as Xyn10RE, Xyn10TC, Xyn10BS, Xyn10PC, and Xyn10C, which hold substantial promise for applications in bioethanol production, animal feeding, and baking. Using the acid stable GH10 Xyn10RE as a model, key amino acids influencing acid stability (nearly 34 residues) were found to be primarily enriched in two specific surface regions behind the enzymatic active center (Supplemental Fig. S2). Furthermore, it is posited that the complex acid stability of GH10 xylanases is jointly determined by surface charges, π-π stacking, ionic bonds, hydrogen bonds, and Van der Waals forces. This research provides gene resources for further application and an essential basis for the acid stable modification of GH10 xylanases.

## Supplementary Information

Below is the link to the electronic supplementary material.

Supplementary file1 (PDF 423 KB)

## Data Availability

All data generated or analyzed during this study are included in this published article (and its supplementary information files).
