# Supplementary material for: Clustered surface amino acid residues modulate the acid stability of GH10 xylanase in fungi
Source: Appl Microbiol Biotechnol. 2024 Feb 16;108(1):216. doi: 10.1007/s00253-024-13045-1 (PMC10873454; doi:10.1007/s00253-024-13045-1)
Supplement: Supplementary file 1 — Supplementary file1 (PDF 423 KB) [file 253_2024_13045_MOESM1_ESM.pdf]

# Supplemental Material

## Applied Microbiology and Biotechnology

**Clustered surface amino acid residues modulate the acid stability of GH10 xylanase in fungi**

Yanwei Xia[1,#], Wei Wang[1,#], Yaning Wei[1], Chuanxu Guo[1], Sisi Song[1], Siqi Cai[1] and Youzhi Miao[1,*]

[1]Jiangsu Provincial Key Lab for Organic Solid Waste Utilization, National Engineering Research Center for Organic-based Fertilizers, Jiangsu Collaborative Innovation Center for Solid Organic Waste Resource Utilization, Nanjing Agricultural University, Nanjing, 210095, China.

[#]These authors contribute equally to this article.

[*]Address correspondence to: Youzhi Miao, yzmiao@njau.edu.cn

Mailing address: College of Resources & Environmental Science, Nanjing Agricultural University, 210095, Nanjing, China, Tel: 86-25-84396477; Fax: 86-25-84396260;

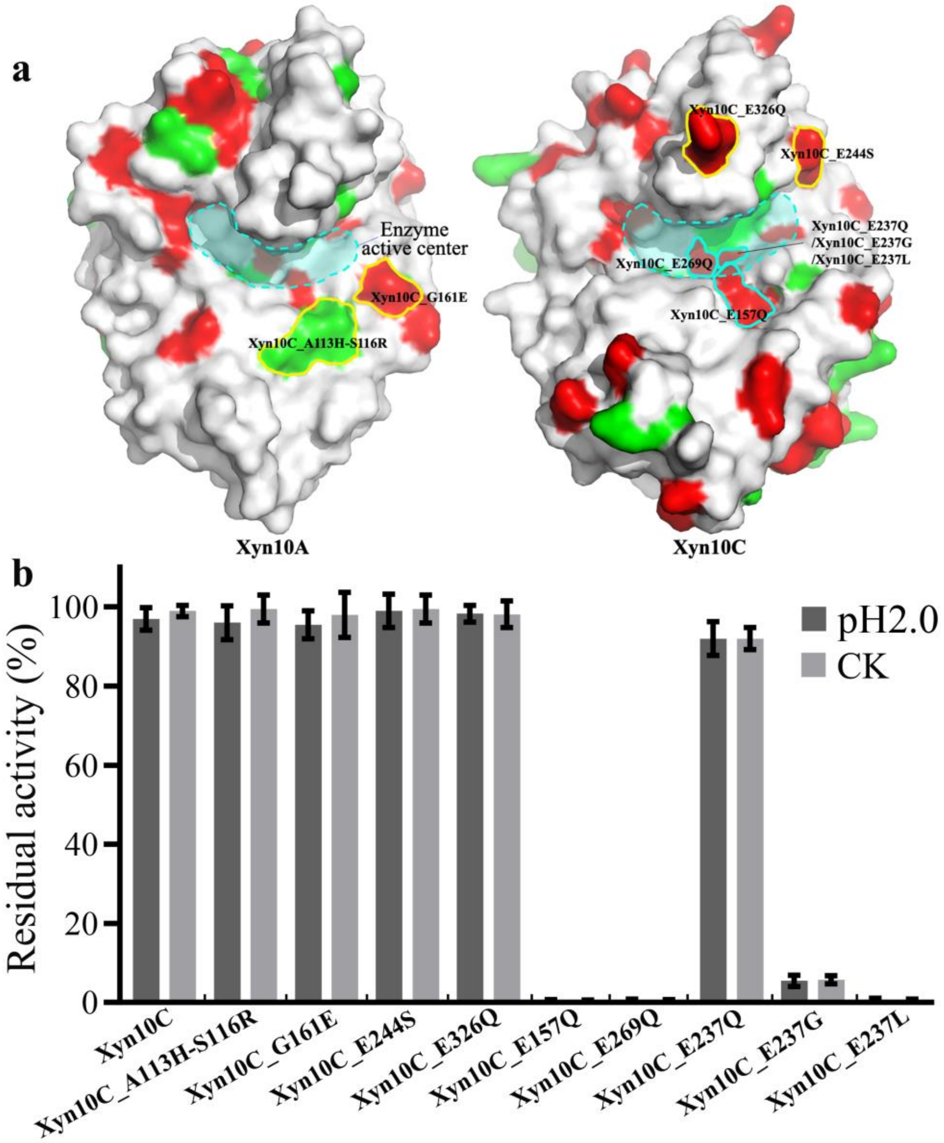


**Fig. S1 Detecting the acid stability of Xyn10C and its different mutants at pH 2.0.** The color of red and green, respectively, on the protein structures of Xyn10A and Xyn10C, corresponds to the acidic residues (D and E) and the basic residues (R, K and H) (**a**). By comparison, a total of 7 different residues located in or around the enzyme active center were used to construct 9 Xyn10C mutants (Xyn10C_A113H-S116R, Xyn10C_G161E, Xyn10C_E244S and Xyn10C_E326Q, Xyn10C_E237Q, Xyn10C_E237G, Xyn10C_E237L, Xyn10C_E157Q and Xyn10C_E269Q). The residual xylanase activity of Xyn10C and these mutants was detected at the optimal conditions after incubation at pH 2.0 for 1 h (**b**). CK means a direct detection without acid treatment.

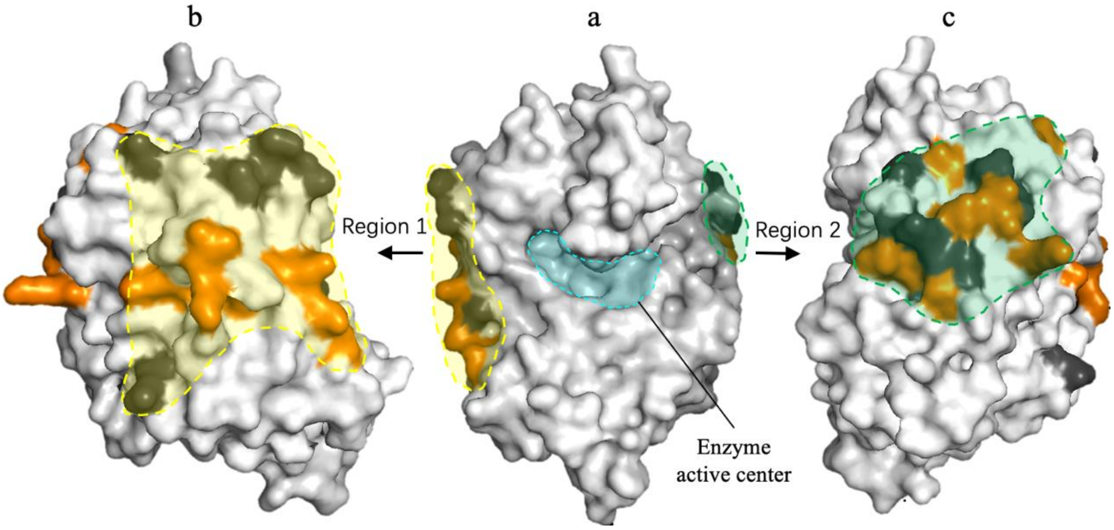


**Fig. S2 Key amino acid residues influencing acid stability of Xyn10RE.** A total of 34 amino acids, including all substitutions in the Xyn10RE_9, Xyn10RE_11, and Xyn10RE_12 mutants (refer to **Supplemental Table S3**), are predominantly located on the backside of the Xyn10RE's 3D structure, opposite the enzyme active center (**a**). These residues form two distinct areas: region 1 (**b**) and region 2 (**c**). The brown and grey colors in the figure represent the amino acid residues of Group A and Group B, respectively.

## Table S1 The enzymatic properties of different GH10 xylanases.

| | | Xyn10A | Xyn10AR (EYE97139.1) | Xyn10AO (AFP43760.1) | Xyn10PR (CDM37719.1) | Xyn10PP (AVA16958.1) | Xyn10TC (BAN82655.1) | Xyn10RE (CAD34597.1) | Xyn10BS (ACS96449.1) | Xyn10PC (ACP27611.1) |
|---|---|---|---|---|---|---|---|---|---|---|
| Clusters | | I | II | II | III | nc | IV | IV | IV | IV |
| Optimal Temperatures | | 90℃ | 80℃ | 80℃ | 80℃ | 75℃ | 70℃ | 80℃ | 85℃ | 70℃ |
| Optimal pH | | 6.0 | 5.0 | 6.0 | 4.0 | 4.0 | 4.0 | 4.0 | 6.0 | 5.0 |
| Specific activity ($U\cdot mg^{-1}$) | | 553.6±36.4 | 168.5±16.5 | 236.1±17.8 | 676.5±14.8 | 407.9±21.3 | 425.9±12.7 | 386.5±12.9 | 938.0±5.9 | 245.9±17.5 |
| $K_m$ value ($mg\cdot mL^{-1}$) | | 11.3±1.8 | 13.7±2.9 | 24.3±2.3 | 14.7±1.6 | 12.1±1.8 | 23.4±2.6 | 56.9±6.3 | 8.38±0.6 | 15.1±3.4 |
| Residual activities after incubation at 70℃ | 10 min | 87.8±3.6 | 73.8±1.5 | 83.2±3.2 | 71.9±0.9 | 79.1±3.2 | 36.1±7.8 | 92.2±1.4 | 90.9±2.5 | 11.2±0.9 |
| | 30 min | 69.2±1.1 | 48.6±2.6 | 62.1±1.4 | 33.9±1.6 | 39.0±3.4 | 24.9±1.1 | 67.3±2.0 | 75.1±0.4 | 2.3±0.4 |
| | 1 h | 37.1±2.8 | 21.3±0.7 | 30.9±0.3 | 10.3±0.6 | 11.2±0.6 | 10.4±5.7 | 44.6±0.8 | 67.7±0.7 | 0.0±0.0 |
| | 3 h | 11.4±1.3 | 1.5±0.4 | 4.8±0.7 | 0.0±0.0 | 0.4±0.7 | 4.8±1.3 | 5.2±0.8 | 63.5±0.7 | 0.0±0.0 |
| | 6 h | 1.3±0.7 | 0.0±0.0 | 0.7±0.4 | 0.0±0.0 | 0.0±0.0 | 1.0±0.5 | 1.2±0.2 | 52.1±0.4 | 0.0±0.0 |

For these enzymes, both optimal temperature and thermostability are assessed at each optimal pH, while optimal pH is determined at each optimal temperature. nc, no cluster.

## Table S2 The distribution of *π-π* stacking types between GH10 xylanases in different clusters.

| **Clusters** | **Names** | ***π-π* stacking types** | | | | | | | | |
|---|---|---|---|---|---|---|---|---|---|---|
| | | His-Phe | His-Trp | His-Tyr | Phe-Phe | Phe-Trp | Phe-Tyr | Trp-Trp | Trp-Tyr | Tyr-Tyr |
| **I** | Y699_04481 (**Xyn10A**) | 2 | 4 | 0 | 0 | 4 | 3 | 3 | 3 | 3 |
| | KEY79926.1 | 2 | 4 | 0 | 0 | 4 | 3 | 3 | 3 | 3 |
| | XP_001258500.1 | 2 | 4 | 0 | 0 | 4 | 3 | 3 | 3 | 3 |
| | GAQ02815.1 | 2 | 4 | 0 | 0 | 4 | 3 | 3 | 3 | 3 |
| | XP_024683552.1 | 2 | 4 | 0 | 0 | 4 | 3 | 3 | 3 | 3 |
| | GAO88483.1 | 2 | 4 | 0 | 0 | 4 | 3 | 3 | 3 | 3 |
| | RLL99221.1 | 2 | 4 | 0 | 0 | 4 | 3 | 3 | 3 | 3 |
| | RHZ56388.1 | 2 | 4 | 0 | 0 | 4 | 3 | 3 | 3 | 3 |
| | XP_026615005.1 | 2 | 4 | 0 | 0 | 4 | 3 | 3 | 3 | 3 |
| **II** | EYE97139.1 (**Xyn10AR**) | 2 | 4 | 0 | 0 | 4 | 2 | 3 | 3 | 4 |
| | ODM22525.1 | 2 | 4 | 0 | 0 | 4 | 2 | 3 | 3 | 4 |
| | AFP43760.1 (**Xyn10AO**) | 2 | 4 | 0 | 0 | 4 | 2 | 4 | 3 | 3 |
| | XP_022484985.1 | 2 | 4 | 0 | 0 | 3 | 3 | 2 | 2 | 4 |
| **III** | XP_022399347.1 | 2 | 4 | 0 | 0 | 4 | 3 | 3 | 3 | 4 |
| | OQD79351.1 | 2 | 4 | 0 | 1 | 3 | 2 | 2 | 2 | 4 |
| | OQE83864.1 | 1 | 4 | 1 | 0 | 3 | 2 | 2 | 2 | 5 |
| | OQE17690.1 | 1 | 4 | 1 | 0 | 3 | 2 | 2 | 2 | 5 |
| | OQE01923.1 | 1 | 4 | 1 | 0 | 3 | 2 | 2 | 2 | 5 |
| | CRL21214.1 | 1 | 4 | 1 | 0 | 3 | 2 | 2 | 2 | 5 |
| | XP_016600950.1 | 1 | 4 | 1 | 0 | 3 | 2 | 2 | 2 | 5 |
| | CDM37719.1 (**Xyn10PR**) | 1 | 4 | 1 | 0 | 3 | 2 | 2 | 2 | 5 |
| | OQE05466.1 | 1 | 4 | 1 | 0 | 3 | 2 | 2 | 2 | 5 |
| | OQE18216.1 | 1 | 4 | 1 | 0 | 4 | 1 | 3 | 3 | 5 |
| **IV** | ACP27611.1 (**Xyn10PC**) | 3 | 3 | 1 | 7 | 1 | 6 | 0 | 1 | 2 |

| | | | | | | | | | | |
|---|---|---|---|---|---|---|---|---|---|---|
| | Y699_06333 (**Xyn10C**) | 3 | 3 | 0 | 6 | 2 | 6 | 0 | 1 | 2 |
| | ACS96449.1 (**Xyn10BS**) | 3 | 3 | 1 | 4 | 3 | 4 | 1 | 2 | 2 |
| | BAN82655.1 (**Xyn10TC**) | 1 | 4 | 1 | 1 | 3 | 3 | 1 | 2 | 7 |
| | CAG25554.1 | 1 | 5 | 1 | 1 | 3 | 4 | 1 | 2 | 7 |
| | CAD34597.1 (**Xyn10RE**) | 2 | 4 | 0 | 0 | 4 | 2 | 3 | 3 | 4 |

**Table S3 The substitution detail and enzymatic properties of all Xyn10RE mutants**

| Names | Substitution details | Specific activity ($U \cdot mg^{-1}$) | $K_m$ value ($mg \cdot mL^{-1}$) | Optimal pH |
|---|---|---|---|---|
| Xyn10RE_1 | I32K,T38S,S47T,T49S,E52V,T53A,N56S,Q59D,L64I,A67G,E78S,V81S,T83S,S85A,A86N,Q89A,I90V,A91V,K95N,A96K,M100L,L101M,N105T,Y109H,N110S,S114N,T117S,E124A,Q144K,D157E,Y161F,S163N,N164S,Y169I,E172P,N188D,A189V,P201S,L211I,L214M,V215I,Q216K,S217A,R221K,S229A,E235S,T239Q,S241D,Q242L,Q243T,Q244T,N245V,M246L,A247K,A248G,F249Y,I258Y,E269S,E271A,L273K,T275A,A278S,Y281F,S283G,T284V,V285A,Q286A,A289V,N290S,K292T,I297V,V299I,T312V,S314Q,D318A,A319P,C320L,A324E,Q327V,E333D,I335L,L336M,T337A | 266.2±11.4 | 4.6±0.3 | 5.0 |
| Xyn10RE_2 | I32K,T38S,T83S,S85A,Q89A,A91V,K95N,A96K,M100L,L101M,N105T,T117S,Q144K,Y169I,P201S,L214M,S229A,Q242L,Q243T,N245V,M246L,A248G,I258Y,L273K,Y281F,S283G,T284V,V285A,N290S,K292T,T312V,S314Q,A319P,C320L,I335L,L336M | 256.3±12.0 | 7.9±1.5 | 5.0 |
| Xyn10RE_3 | 32K,T38S,S47T,T49S,E52V,T53A,N56S,Q59D,L64I,A67G,E78S,V81S,T83S,S85A,A86N,Q89A,I90V,A91V,K95N,A96K,M100L,L101M,N105T,Y109H,N110S,S114N,T117S,E124A,Q144K,D157E,Y161F,S163N,N164S,Y169I,E172P,N188D,A189V | 226.4±5.4 | 2.7±0.1 | 5.0 |
| Xyn10RE_4 | P201S,L211I,L214M,V215I,Q216K,S217A,R221K,S229A,E235S,T239Q,S241D,Q242L,Q243T,Q244T,N245V,M246L,A247K,A248G,F249Y,I258Y,E269S,E271A,L273K,T275A,A278S,Y281F,S283G,T284V,V285A,Q286A,A289V,N290S,K292T,I297V,V299I,T312V,S314Q,D318A,A319P,C320L,A324E,Q327V,E333D,I335L,L336M,T337A | 429.8±16.2 | 5.1±0.5 | 5.0 |
| Xyn10RE_5 | S47T,T49S,E52V,T53A,N56S,Q59D,L64I,A67G,E78S,V81S,A86N,I90V,Y109H,N110S,S114N,E124A,D157E,Y161F,S163N,N164S,E172P,N188D,A189V,L211I,V215I,Q216K,S217A,R221K,E235S,T239Q,S241 | 478.7±17.8 | 33.8±5.8 | 6.0 |

| | | | | |
|---|---|---|---|---|
| | D,Q244T,A247K,F249Y,E269S,E271A,T275A,A278S,Q286A,A289V,I297V,V299I,D318A,A324E,Q327V,E333D,T337A | | | |
| Xyn10RE_6 | I32K,T38S,T83S,S85A,Q89A,A91V,K95N,A96K,M100L,L101M,N105T,T117S,Q144K,Y169I | 290.1±11.2 | 29.6±5.4 | 4.0 |
| Xyn10RE_7 | P201S,L214M,S229A,Q242L,Q243T,N245V,M246L,A248G,I258Y,L273K,Y281F,S283G,T284V,V285A,N290S,K292T,T312V,S314Q,A319P,C320L,I335L,L336M | 180.9±7.3 | 25.1±3.2 | 4.0 |
| Xyn10RE_8 | T83S,S85A,Q89A,A91V,K95N,A96K,M100L,L101M | 421.8±10.5 | 18.7±4.1 | 4.0 |
| Xyn10RE_9 | P201S,Q243T,N245V,M246L,A248G,S283G,N290S,K292T,L336M | 335.0±6.8 | 8.6±1.8 | 5.0 |
| Xyn10RE_10 | T83S,S85A,Q89A,A91V,K95N,A96K,M100L,L101M,P201S,Q243T,N245V,M246L,A248G,S283G,N290S,K292T,L336M | 342.2±11.9 | 26.9±1.9 | 4.0 |
| Xyn10RE_11 | S47T,T49S,E52V,T53A,N56S,Q59D,T83S,S85A,A86N,Q89A,I90V,A91V,K95N,A96K,M100L,L101M,N188D,A189V | 260.5±6.6 | 21.7±5.2 | 4.0 |
| Xyn10RE_12 | T239Q,S241D,Q244T,A247K,F249Y,Q286A,A289V | 277.3±3.5 | 28.1±4.3 | 4.0 |
| Xyn10RE_13 | A67G,E78S,V81S,N105T,Y109H,N110S,S114N,T117S,E124A,D157E,Y161F,S163N,N164S,Y169I,E172P | 223.8±3.6 | 47.2±6.5 | 4.0 |
| Xyn10RE_14 | L214M,V215I,Q216K,S217A,R221K,S229A,I258Y,E269S,E271A,L273K,T275A,A278S,T312V,S314Q,D318A,A319P,C320L,A324E,Q327V,E333D,I335L | 924.9±37.8 | 7.1±0.2 | 6.0 |

**Table S4 The changes of non-covalent interactions in Xyn10RE and its mutants.**

| | Hydrogen bonds | π-π Stack | π-Cation | Ionic bonds | Disulphide bonds | Van der Waals forces |
|---|---|---|---|---|---|---|
| Xyn10RE | 295 | 22 | 3 | 5 | 2 | 330 |
| Xyn10RE_1 | 292 | 22 | 3 | 6 | 2 | 327 |
| Xyn10RE_2 | 289 | 22 | 3 | 6 | 2 | 328 |
| Xyn10RE_3 | 293 | 22 | 3 | 6 | 2 | 313 |
| Xyn10RE_4 | 293 | 22 | 3 | 5 | 2 | 332 |
| Xyn10RE_5 | 293 | 22 | 3 | 6 | 2 | 330 |
| Xyn10RE_6 | 294 | 22 | 3 | 5 | 2 | 317 |
| Xyn10RE_7 | 292 | 22 | 3 | 5 | 2 | 337 |
| Xyn10RE_8 | 294 | 22 | 3 | 5 | 2 | 331 |
| Xyn10RE_9 | 294 | 22 | 3 | 5 | 2 | 327 |
| Xyn10RE_10 | 293 | 22 | 3 | 5 | 2 | 328 |
| Xyn10RE_11 | 294 | 22 | 3 | 5 | 2 | 330 |
| Xyn10RE_12 | 294 | 22 | 3 | 6 | 2 | 323 |
| Xyn10RE_13 | 294 | 22 | 3 | 5 | 2 | 334 |

| | | | | | | |
|---|---|---|---|---|---|---|
| Xyn10RE_14 | 294 | 22 | 3 | 5 | 2 | 327 |
